# miR-338-3p Plays a Significant Role in Casticin-Induced Suppression of Acute Myeloid Leukemia via Targeting PI3K/Akt Pathway

**DOI:** 10.1155/2022/9214130

**Published:** 2022-06-18

**Authors:** Kewei Yu, Juan Wang, Junhui Hou, Lei Zhang, Hui Liang

**Affiliations:** ^1^Department of Pharmacy, Jinan Central Hospital, Cheeloo College of Medicine, Shandong University, Jinan, 250013 Shandong, China; ^2^Department of Gynaecology, Jiyang People's Hospital, Jinan 251400, China; ^3^Department of Tumor Radiotherapy (III), The Affiliated Qingdao Central Hospital of Qingdao University, The Second Affiliated Hospital of Medical College of Qingdao University, Qingdao 266042, China; ^4^Department of Hematology, Qingdao Women and Children's Hospital, Qingdao 266034, China

## Abstract

**Objective:**

Casticin is generally used in traditional herbal medicine for its anti-inflammatory and anticarcinogenic pharmacological properties. Also, microRNAs are indispensable oncogenes or cancer suppressors being dysregulated in various diseases. In this study, we aimed to elucidate the mechanisms underlying effects of casticin on the progression of acute myeloid leukemia (AML).

**Methods:**

CCK-8 and flow cytometry were utilized to measure the proliferation and apoptosis of AML cell lines, respectively, after treatment with different concentrations of casticin. The alteration of several microRNA expressions in response to casticin treatment was detected by performing qRT-PCR, and the activity of PI3K/Akt pathways was evaluated through immunoblotting. Afterwards, the potential target gene of miR-338-3p was investigated by dual-luciferase reporter assay. In order to evaluate the role of miR-338-3p in the casticin-induced cellular phenotype changes, AML cells were transfected with miR-338-3p mimics or inhibitor and then subjected to proliferation and apoptosis analysis. Finally, a mouse xenograft model system was employed to investigate the role of casticin in AML progression in vivo.

**Results:**

Suppressed cellular proliferation and enhanced apoptosis were observed in HL-60 and THP-1 cells after exposure to casticin, accompanied by remarkable upregulation of the miR-338-3p expression as well as a decline in the phosphorylation of PI3K and Akt proteins. RUNX2 was identified as a direct target molecular of miR-338-3p, which might account for the findings that miR-338-3p knockdown enhanced the PI3K/Akt pathway activity, whereas the miR-338-3p overexpression inactivated this signaling pathway. In addition, the inhibition of the miR-338-3p expression attenuated severe cell apoptosis and suppressions of PI3K/Akt pathway induced by casticin. Furthermore, casticin treatment retarded tumor growth rate in mouse models, whilst elevating miR-338 expression and repressing the activity of PI3K/Akt pathway in vivo. However, miR-338-3p depletion could also abolish the phenotypic alterations caused by casticin treatment.

**Conclusion:**

Casticin promotes AML cell apoptosis but inhibits AML cell proliferation in vitro and tumor growth in vivo by upregulating miR-338-3p, which targets RUNX2 and thereafter inactivates PI3K-Akt signaling pathway. Our results provide insights into the mechanisms underlying the action of casticin in the control of AML progression.

## 1. Introduction

Acute myeloid leukemia (AML) is an aggressive clonal disorder of the hematopoietic system characterized by cytogenetic heterogeneity, which manifests as malignant proliferation of immature myeloid progenitor cells and impaired generation of normal hematopoietic stromal cells [1]. AML occurs in ~80% of adult patients with acute leukemia [2]. It progresses rapidly, and most patients will have relatively poor clinical outcomes. Typically, patients with AML initially receive intensive induction therapy, followed by additional chemotherapy, targeted therapy, or stem-cell transplantation for postremission consolidation [3]. Less than half of these patients achieve long-time survival, and the others will die of this disease owing to the occurrence of relapse [4]. Therefore, it is of great realistic significance to develop novel effective therapeutic methods to treat the refractory AML.

Accumulating evidence have demonstrated that chemicals derived from traditional Chinese medicine can be used for treatment of human aliments [5–9]. Casticin, a bioactive ingredient isolated from the plant *Vitex rotundifolia*, is well known for its anti-inflammatory bioactivity and has been extensively applied in traditional Chinese medicine for a long time [10]. Increasing number of studies have reported that casticin can induce cell cycle arrest and apoptosis of cancerous cells in various kinds of carcinomas including melanoma [11], bladder cancer [12], cervical cancer [13], breast cancer [14], and colon cancer [15], and this may probably account for its anticarcinogenic actions. It has been observed that casticin exerts its synergistic effects when used in accompany with other chemotherapeutic drugs [16]. Apart from anti-inflammatory and anticarcinogenic activities, casticin possesses diverse functions such as immunoregulation, neuroprotection, and antihyperprolactinemia, as well as analgesia [17]. In particular, the apoptosis-promoting activity of casticin has been also found in leukemic cells [18, 19], but the detailed molecular mechanisms underlying how casticin plays its role in the suppression of leukemia development are still incompletely understood. Previous study proposed that casticin effectively promotes cell apoptosis in K562 cells by blocking PI3K/Akt pathway that is implicated in the regulation of cell survival [18]. In fact, suppression of Akt phosphorylation can enhance cellular apoptosis in AML [20], and a variety of antileukemia drugs may act, at least in part, by inactivating Akt protein [21].

MicroRNAs are defined as a class of noncoding and small RNA molecules (encompassing approximately 22 nucleotides) that can inhibit gene expression at posttranscriptional level by interacting with 3′-UTR of the target gene, contributing to either degradation or translational suppression of mRNAs [22]. An increasing number of studies have revealed that miRNAs are indispensable oncogenes or cancer suppressors being dysregulated in AML development [23–25], suggesting that miRNAs may serve as potential pharmaceutical targets for AML therapy.

In this context, we presently evaluated the biological behaviors in HL60 cell and xenograft tumor growth after exposure to casticin treatment and also investigated the potential involved microRNAs and intracellular pathway. Our results will further improve our understanding about how casticin affects cell growth in the AML pathogenesis.

## 2. Materials and Methods

### 2.1. Cell Culture

Human promyelocytic leukemia cell line HL-60 and normal bone marrow cell line (HS-5) were kindly given by the Oncology Center of Shandong University (Jinan, Shandong, China). The gifted cell line was approved by the Ethics Committee of Qianfoshan Hospital, affiliated to Shandong University. Another AML cell line THP-1 was purchased from American Type Culture Collection (ATCC). Cells were grown in RMPI 1640 medium (Gibco, Gaithersburg, MD, USA) supplemented with 10% fetal bovine serum (FBS, Gibco) and 1% penicillin-streptomycin and maintained at 37°C in a humidified atmosphere of 5% CO_2_.

### 2.2. Preparation of Casticin Reagent

Casticin was provided by the laboratory of medical engineering, Hunan Normal University (Changsha, Hunan, China), and the purity achieved 99%. Casticin was then dissolved in 10% dimethylsulfoxide (DMSO) in PBS and added into RMPI 1640 medium at the final concentrations of 1, 2, 4, and 8 g/mL. Cells were starved for 16 h before casticin treatment.

### 2.3. CCK8 Assay

Cell proliferation was assessed using the Cell Counting Kit-8 (Dojindo Laboratories, Gaithersburg, MD, USA) according to the manufacturer's protocols. In brief, cells (5 × 10^4^ cells/well) were plated into 96-well plates and were incubated at 37°C before they were exposed to escalating concentrations of casticin (1, 2, 4, 8 mg/mL) stepwise. At the indicated time points, 10 *μ*L of CCK-8 solution was added to each well, and the cells were incubated for another 2 ~ 4 h. The absorbance of samples was measured at 495 nm using a microplate reader (Bio-Rad, Hercules, CA, USA).

### 2.4. Cell Apoptosis Analysis

Annexin V-fluorescein isothiocyanate (FITC) apoptosis detection kit (BD Biosciences, San Jose, CA, USA) was used to assess the apoptosis rate of HL-60 cells. Cells were washed twice with prechilled PBS, centrifugated and resuspended in binding buffer, and then incubated with 5 *μ*L of annexin V-FITC for 30 min in dark. Next, cells were incubated with 5 *μ*L of PI for 5 min in dark before they were subjected to analysis with a flow cytometer (FACScan, BD Biosciences).

### 2.5. Quantitative RT-PCR

Total RNA was extracted from AML cells using TRIzo Reagent (TaKaRa, Dalian, China). Afterwards, cDNA was generated from 1 *μ*g RNA as template by performing reverse transcription PCR. miR-338-3p was quantified by virtue of a TaqMan miRNA Reverse-Transcription Kit (Life Technologies, Carlsbad, CA, USA), and U6 snRNA acted as the internal control. The gene expression of *RUNX2* was assessed by using One Step Prime Script miRNA cDNA Synthesis Kit (Qiagen, Valencia, CA, USA) and a SYBR™ PrimeScript RT-PCR Kit (TaKaRa), according to the manufacturer's instructions. Primer sequences were shown in Table 1. The comparative 2 − ^*ΔΔ*Ct^ method was used for relative quantification and statistical analysis.

### 2.6. TUNEL Staining

Apoptotic cells were detected using the in situ cell death detection kit (Roche, Mannheim, Germany) following the manufacturer's protocol. TUNEL staining–positive cells in the cartilage area were visualized under a fluorescence microscope.

### 2.7. Cell Transfection

The miR-338-3p mimic, inhibitor, and corresponding negative control (NC) were purchased from GenePharma (Shanghai, China). 50 nM miR-338-3p/NC mimics or inhibitor was transfected into cells (2 × 10^5^/well) seeded in a 6-well plate, using Lipofectamine 3000 reagent (Invitrogen, Grand Island, NY, USA) according to the manufacturer's protocol. Cells were incubated at 37°C with 5% CO_2_ and harvested at least 48 h later for the subsequent study.

### 2.8. Dual Luciferase Reporter Assay

The human RUNX2 3′-UTR fragment containing the wild-type or mutant miR-338-3p binding site was synthesized by Invitrogen and amplified before cloned into pmirGLO Vector (Promega, Madison, WI, USA) between the SacI and XhoI sites. Cells were subsequently cotransfected with miR-338-3p (or miR-NC) and the reconstructed vector and subjected to luciferase activity analysis using the dual-luciferase assay system (Promega) after 48 h transfection. Renilla-luciferase activity was used for normalization.

### 2.9. Western Blotting

Primary antibodies against RUNX2, *β*-actin, p-Akt, Akt, p-PI3K, PI3K, and HRP-conjugated goat anti-rabbit IgG were purchased from Cell Signaling Technology (Beverly, MA, USA). Proteins were extracted from cell lysates with RIPA lysis buffer (Beyotime Biotech, Shanghai, China) following the manufacturer's instructions. The total proteins were resolved by SDS-PAGE (10% acrylamide) before being transferred to PVDF membrane, which were blocked with 5% silk milk for at least 1 h and incubated overnight at 4°C with specific primary antibodies. After rinsing with TBST, the membrane was then incubated for 1 h at room temperature with goat anti-rabbit IgG. Finally, the labeled proteins were visualized with ECL substrates (Millipore, MA, USA) before quantifying the band intensity using Image Lab TM software (Bio-Rad, Shanghai, China).

### 2.10. Animal Experiment

4 ~ 6-week-old BALB/c nude mice were purchased from the Provincial Laboratory Animal Center (Jinan, Shandong, China) and maintained on a 12-h light–dark cycle and were allowed free access to standard rodent chow and water ad libitum. All the mice were randomly divided into groups: (1) AML model (as the control group), (2) casticin, (3) miR-338 inhibitor, and (4) casticin + miR-338 inhibitor, *n* = 5 in each group. 5 × 10^6^ HL-60 cells were subcutaneously injected into mice to obtain AML mouse models, and for groups (3) ~ (4), HL-60 cells transfected with miR-338 were used. Additionally, mice were administrated with casticin (40 mg/kg/d) to evaluate its antitumor role in vivo, while the control mice received only normal saline vehicle. The width and length of tumor xenografts were detected at different time points to calculate tumor volumes: volume = width^2^ × length/2. After 4 weeks, the mice were sacrificed, and tumor xenografts were weighed and analyzed for gene and protein expression. This experiment was approved by the Ethics Committee of Qianfoshan Hospital, in accordance with the guidelines of our hospital regarding the welfare and ethic for experimental animals.

### 2.11. Statistical Analysis

Data are presented as means ± SD. All experiments included at least 3 replicates per group. Groups were compared using the two-tailed Student's *t*-test for parametric data. All statistical analyses were carried out using the SPSS 19.0 software (SPSS, Inc., Chicago, IL). A value of *P* < 0.05 was considered statistically significant.

## 3. Results

### 3.1. Casticin Induces the Apoptosis of HL-60 Cells

**Figure 1:**
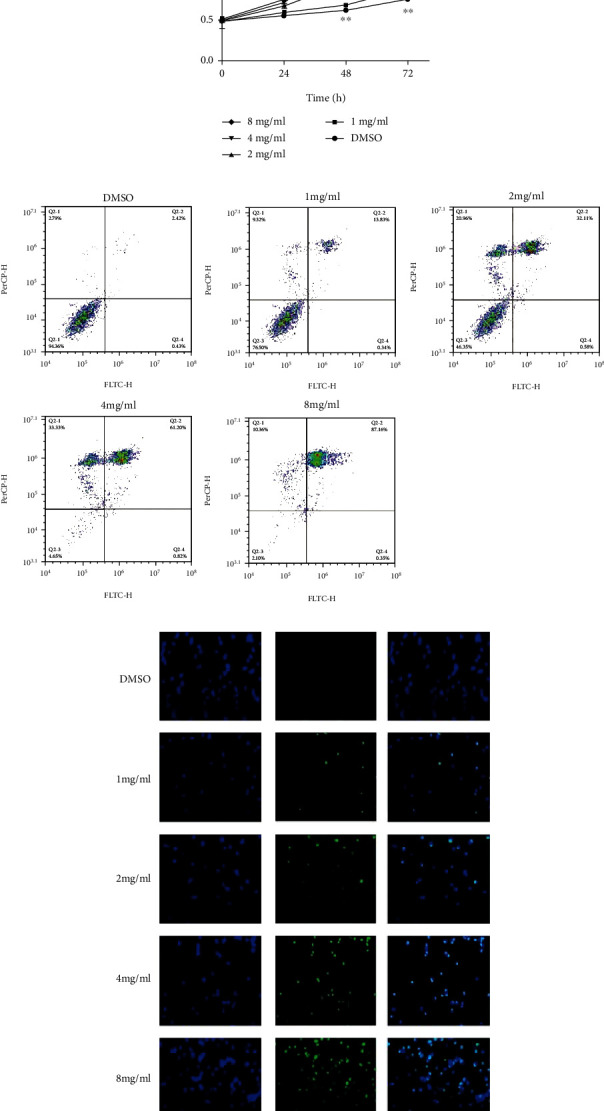
Casticin inhibits HL-60 cell proliferation and promotes apoptosis. (a) HL-60 cells were treated with different concentrations of casticin (1, 2, 4, 8 mg/mL) and subjected to CCK8 analysis at the indicated time intervals. After treatment with casticin for 72 h, cell apoptosis was analyzed by performing flow cytometry (b) and TUNEL assay (c). Compared with the DMSO control group, ^∗^ indicates *P* < 0.05; ^∗∗^ indicates *P* < 0.01.

After human promyelocytic leukemia HL-60 cells were treated with casticin, there was a significant decline in cell viability (Figure 1(a)), and the impact of casticin on cell viability was in a dose- and time-dependent manner. Subsequently, flow cytometry was performed to analyze the apoptosis rate of HL-60 in response to casticin treatment, which demonstrated a dramatic increase in the proportion of apoptotic cells in parallel with the augmented casticin concentration (Figure 1(b)).

Additionally, we found that the number of TUNEL-stained HL-60 cells was increased when cells were treated with higher concentration of casticin (Figure 1(c)), consistent with results in Figure 1(b). These all indicate that casticin inhibits the proliferation and enhances apoptosis in HL-60 cells in a dose-dependent manner. In order to confirm these results, another AML cell line THP-1 was subjected to casticin treatment. We observed that casticin not only mitigated THP-1 cell proliferation but also induced cells to undergo apoptosis in a dose- and time-dependent manner (Figure S1).

### 3.2. miR-338-3p Is Upregulated after Casticin Treatment

**Figure 2:**
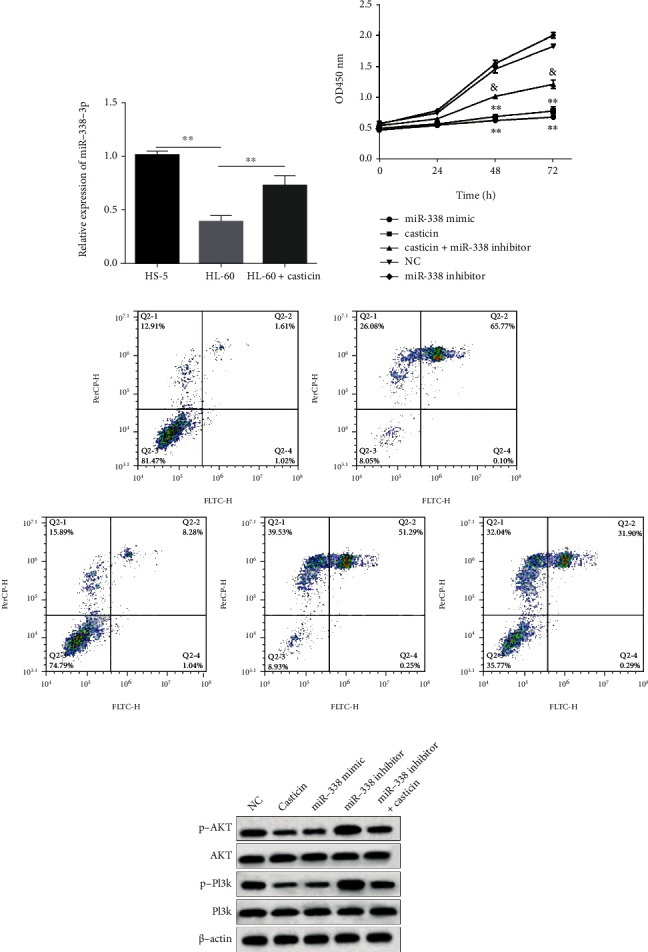
miR-338-3p is upregulated after casticin treatment, and miR-338-3p silencing mitigates the effect of casticin on PI3K/Akt activity. HL-60 cells were treated with 8 mg/mL casticin for 72 h. (a) The miR-338-3p expression was measured by qRT-PCR. ^∗∗^*P* < 0.01. (b) CCK8 was used to assess the viability of HL-60 cells transfected with miR-338-3p mimics or inhibitor. (c) Flow cytometry was carried out to test the apoptosis of HL-60 cells. ^∗^*P* < 0.05 and ^∗∗^*P* < 0.01 compared with the NC group, and “&” indicates *P* < 0.05 compared to untransfected cells treated with casticin. (d) Expression of the major components in PI3K/Akt pathway and the phosphorylated forms of them were determined using immunoblotting. NC: untransfected cells used as the negative control.

n order to further determine how casticin affects the phenotype of AML cells, we next detected the expression alteration of several microRNAs in HL60 and THP-1 cells after exposure to casticin. These microRNAs were previously reported to show aberrant expression in AML patients, which is associated with different prognoses of this disease [23–25], including miR-29, miR-338, miR-125, miR-142, miR-146, and miR-155 (data not shown), but among them, only miR-338 was sensitive to casticin treatment. As shown in Figure 2(a) and Figure S1-S2, miR-338-3p was suppressed in both HL60 and THP-1 compared with the normal bone marrow cells but obviously upregulated after exposure to casticin (*P* < 0.01), which was dose-dependent.

### 3.3. miR-338-3p Depletion Attenuates the Casticin-Induced Apoptosis in HL60 Cells

In order to confirm the role of miR-338-3p in casticin-mediated cell apoptosis in AML, we introduced miR-338-3p mimics or inhibitor into HL-60 cells in prior to casticin treatment. In fact, miR-338-3p has been extensively reported to inhibit the cell proliferation and promote cell apoptosis in various cancers [26–29]. Our results indicated that exogenous miR-338-3p suppressed cell viability and promoted apoptosis of HL-60 cells, whereas the transfection of miR-338-3p inhibitor caused the opposite results (Figures 2(b) and 2(c)). Although casticin dramatically changed cell phenotype as mentioned above, the miR-338-3p inhibitor transfected into HL-60 cells caused an increase in cell viability but impaired apoptosis rate, which seems to abrogate the function of casticin.

### 3.4. Inhibition of miR-338-3p Mitigates the Effect of Casticin on PI3K/Akt Activity

Casticin was demonstrated to inactivate PI3K/Akt signaling pathway thereby enhancing apoptosis in K562, another AML cell line [13]. We therefore evaluated the PI3K/Akt activity in HL60 cells and found that this pathway was distinctly prohibited in the presence of casticin (Figure 2(d)). In addition, aberrant activation of PI3K/Akt pathway was observed in HL60 cells where miR-338-3p was silenced, while it was inactivated in cells with the miR-338-3p overexpression. Intriguingly, when those casticin-treated HL60 cells were subjected to miR-338-3p knockdown, the casticin treatment seemed noneffective.

### 3.5. miR-338-3p Directly Targets RUNX2 in AML Cells

**Figure 3:**
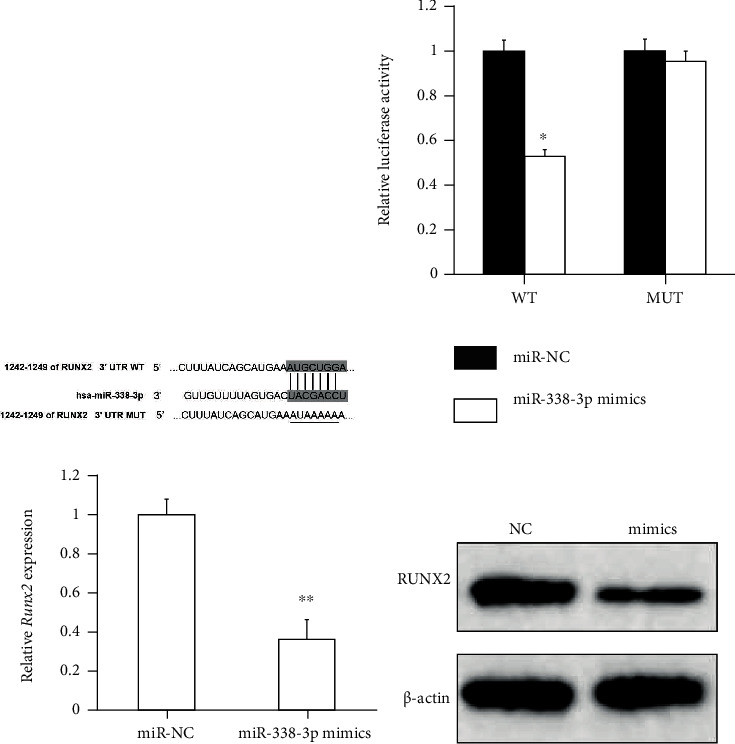
miR-338-3p directly targets RUNX2 in HL-60 cells. (a) The putative binding sites for miR-338-3p in the RUNX2 3′-UTR. WT: wild-type; MUT: mutant sequence. (b) The luciferase activity was detected using the dual luciferase reporter assay, after HL-60 cells were cotransfected with a luciferase plasmid harboring RUNX2 3′-UTR (WT or MUT) and miR-338 mimics or miR-NC. (c, d) The endogenous expression of RUNX2 was evaluated at mRNA and protein levels by conducting RT-qPCR and Western blot assay, respectively. ^∗^ indicates *P* < 0.05; ^∗∗^ indicates *P* < 0.01.

The putative target genes of miR-338-3p were predicted by scanning TargetScan and RegRNA as public miRNA databases. Among numerous candidate genes, RUNX2 harboring a highly conserved binding site in the 3′-UTR for miR-338-3p (Figure 3(a)) was particularly concerned due to its involvement in PI3K/Akt signaling [23]. We next performed luciferase reporter assay to verify the relationship between miR-338-3p and RUNX2. A luciferase reporter plasmid containing the 3′-UTR-binding site of RUNX2 was cotransfected with miR-338-3p response element into HL-60 cells. As shown in Figure 3(b), miR-338-3p mimics impaired the luciferase activity of reporter plasmid carrying wild-type (WT) RUNX2 3′-UTR but had no effect on the mutant (MUT) RUNX2 3′-UTR.

Afterwards, we determined whether miR-338-3p has an impact on the endogenous expression of RUNX2 in HL-60 cells by conducting RT-qPCR and Western blot assay. The results showed that the enhanced expression of miR-338-3p significantly suppressed the RUNX2 expression at both mRNA and protein levels, whereas restriction of miR-338-3p led to an increase in the RUNX2 expression (Figures 3(c) and 3(d)). Taken together, these data demonstrate that miR-338-3p decreases the RUNX2 expression by directly targeting its 3′-UTR in AML cells.

### 3.6. Casticin Controls Tumor Growth In Vivo by Promoting miR-338-3p Expression

**Figure 4:**
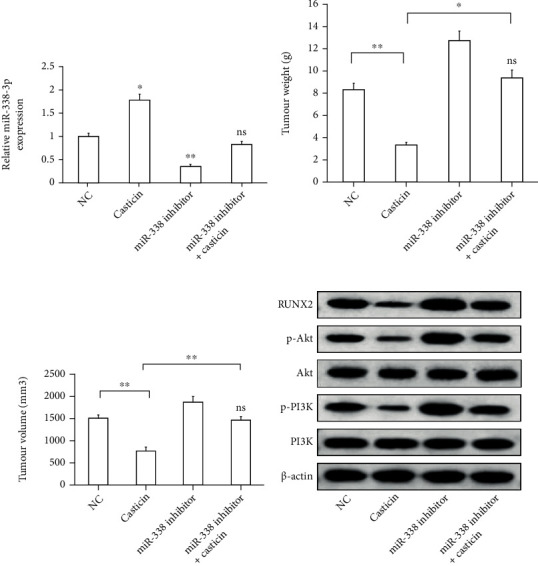
Casticin controls tumor growth in vivo by promoting miR-338-3p expression. HL-60 cells were transfected with miR-338 inhibitor, in the presence or absence of casticin. (a) The expression of miR-338-3p in HL-60 cells undergoing different treatment was assessed by qRT-PCR. Compared to untreated cells, ^∗^*P* < 0.05; ^∗∗^*P* < 0.01; ns means the difference is not significant. The xenograft tumors formed were excised after 4 weeks. (b) The tumor was massed in each group. ^∗^*P* < 0.05; ^∗∗^*P* < 0.01 between two groups; “ns” indicates not significant vs. control group. (c) The width and length of tumor were measured, and tumor volume was determined according to them: volume = width^2^ × length/2. ^∗^*P* < 0.05; ^∗∗^*P* < 0.01 between two groups; “ns”: not significant vs. control group. (d) The expression level of proteins in the tumor xenografts was detected by western blot assay. Beta-actin was used for the normalization.

The effect of casticin on AML progression in vivo was also investigated in mice. Firstly, HL-60 cells were subcutaneously inoculated into the mice, administrated with casticin, and the expression level of miR-338-3p was detected. Of note, according to RT-qPCR analysis, miR-338-3p was upregulated in tumor xenografts derived from casticin-treated HL-60 cells (Figure 4(a), *P* < 0.05). Next, the tumor weight and volume of xenografts were tested to evaluate the effect of casticin on tumor growth in vivo. As illustrated in Figure 4(b), the weight of tumor xenografts in the casticin group was substantially reduced in comparison with the xenografts derived from untreated HL-60 cells. Also, the tumor volume was decreased in the casticin group compared to the NC group (Figure 4(c)).

To confirm that miR-338-3p upregulation was responsible for the inhibitory role of casticin in tumor growth, cells were transfected with miR-338-3p inhibitor before they were subjected to casticin exposure. As shown in Figures 4(b) and 4(c), although the tumor formation was hindered when HL-60 cells were stimulated with casticin, miR-338-3p inhibition could accelerate the process of tumorigenesis in spite of the preceding casticin treatment, suggesting the role of miR-338-3p in the casticin-mediated tumor suppression.

The expression of RUNX2 at the protein level and the activity of PI3K/Akt pathway were analyzed. Compared with the control group, both RUNX2 expression and the phosphorylation of Akt and PI3K proteins were enhanced in the miR-338-3p group. Furthermore, remarkable decrease in the protein levels of RUNX2, p-Akt, and p-PI3K was observed in the xenografts treated with casticin, but these decreased expression profiles were abolished by the addition of miR-338 inhibitor (Figure 4(d)). The results manifest that casticin inhibits AML progression in vivo by upregulating miR-338-3p via RUNX2/PI3K/Akt axis.

## 4. Discussion

Nowadays, the mainstream therapeutic regimens for patients with AML are presented by stem-cell transplantation, chemotherapy, and targeted therapy. Given that chemotherapy cannot cure a majority of AML patients, stem-cell transplantation acts as an alternative treatment strategy in most cases. However, allogeneic stem-cell transplantation is not always feasible for those patients who have developed high-risk leukemia, and therefore, the recurrent and mortality rates in AML patients are still very high. Casticin has been reported to induce the apoptosis of several leukemic cell lines [18, 19], thereafter emerging as promising treatment that may improve the outcomes of AML patients. In this study, after human promyelocytic leukemia HL-60 cells treated with casticin, there was a significant decline in cell viability, and the impact of casticin on cell viability was in a dose- and time-dependent manner. Subsequently, flow cytometry demonstrated a dramatic increase in the proportion of apoptotic cells in parallel with the augmented casticin concentration.

Aberrant alterations in miRNA expression level are tightly related to the initiation and/or progression of diverse malignancies including AML [23–25], which suggests that miRNAs may function as potential targets for AML treatment. miR-338-3p functions as a tumor suppressor in multiple carcinomas by targeting diverse genes and certain pathways. For example, miR-338-3p targets PREX2a, a protein that activates PI3K/Akt signaling by confronting PTEN in cancerous cells, and thus suppresses gastric cancer and neuroblastoma [26, 27]. In our present study, the expression pattern of miR-338-3p was changed upon casticin incitement. Casticin inhibited the proliferation and induced apoptosis in HL-60 cells; therefore, we attempt that casticin affects the phenotype of AML cell through regulating miR-338-3p expression level. ATF2 was also identified as a target gene of miR-338-3p, and it could inhibit the proliferation of cervical cancer cells via targeting the PI3K/Akt/mTOR signaling cascade [28].

Actually, casticin has been reported to enhance apoptosis in K562 cell line by inactivating PI3K/Akt signaling pathway [18], a critical axis modulating cancer cell survival and metastasis. We evaluated the activity of PI3K/Akt pathway in HL-60 cells after casticin treatment and found that the phosphorylated PI3K and Akt proteins were decreased in response to casticin, which is consistent with previous study. Moreover, miR-338-3p knockdown enhanced PI3K/Akt signaling pathway and abrogated the casticin-mediated inactivation of this pathway, whereas the overexpression of miR-338-3p further suppressed PI3K/Akt activity.

On the other hand, we also confirmed that RUNX2 was directly targeted by miR-338-3p in HL-60 cells. RUNX2 is a pivotal regulator for skeletal development but abnormally expressed during carcinogenesis and, in particular, its function as the direct target of miR-338-3p has been previously proved in the ovarian epithelial carcinoma [29]. There is a mutual interaction between RUNX2 and PI3K/Akt pathway: Akt can directly or indirectly enforce RUNX2 expression level and/or transcriptional activity, which will reciprocally contribute to the activation of PI3K/Akt signaling [30]. Therefore, we propose that miR-338-3p may regulate AML cell proliferation and apoptosis in vitro by targeting RUNX2-PI3K/Akt axis.

Additionally, in order to investigate the role of casticin in AML progression in vivo, we employed a mouse xenograft model system, where there was an observation that inhibition of the miR-338-3p expression remarkably accelerated tumorigenesis and abolished the inhibitory effect of casticin on tumor growth.

## 5. Conclusion

In aggregate, our results demonstrate that the miR-338 expression in HL-60 cells was significantly upregulated in response to casticin treatment, companied by enhanced cell apoptosis and PI3K/Akt inactivation. RUNX2 was also identified as the direct target of miR-338 in HL-60 cells. Furthermore, the enhanced miR-338 expression could mimic the inhibitory effect of casticin on cell proliferation and PI3K/Akt pathway activation, whereas miR-338 inhibition mitigated the function of casticin both in vitro and in vivo, suggesting that casticin might suppress AML development and/or progression by increasing the miR-338 expression and targeting RUNX2-PI3K/Akt signaling pathway.

## Figures and Tables

**Table 1:** Primers for reverse transcription (RT) and real-time PCR.

Gene		Sequences
miR-338-3p	Forward	ATCCAGTGCGTGTCGTG
	Reverse	TGCTTCCAGCATCAGTGAT
(for RT)		GTCGTATCCAGTGCGTGTCGTGGAG TCGGCAATTGCACTGGATACGACCAACAAA
U6	Forward	GCTTCGGCAGCACATATACTAAAAT
	Reverse	CGCTTCACGAATTTGCGTGTCAT
(for RT)		CGCTTCACGAATTTGCGTGTCAT
RUNX2	Forward	CTAGGCGCATTTCAGGTGCT
	Reverse	TGGCAGGTAGGTGTGGTAGT
GAPDH	Forward	GCCAAAAGGGTCATCATCTC
	Reverse	GTAGAGGCAGGGATGATGTTC

## Data Availability

The datasets during the current study are available from the corresponding author on reasonable request.

## Supplementary Materials

Supplementary 1 Figure S1: casticin inhibits THP-1 cell proliferation and promotes apoptosis. (a) THP-1 cells were treated with different concentrations of casticin (1, 2, 4, 8 mg/mL) and subjected to CCK-8 analysis at the indicated time intervals. Compared with the DMSO control group, ^∗^ indicates *P* < 0.05; ^∗∗^ indicates *P* < 0.01. (b) After treatment with casticin for 72 h, cell apoptosis was analyzed by performing flow cytometry. (c) Verification of the flow cytometry results by TUNEL assay. ^∗^*P* < 0.05; ^∗∗^*P* < 0.01. (d) The miR-338-3p expression was measured by qRT-PCR in THP-1 cell. ^∗∗^*P* < 0.01.

Supplementary 2 Figure S2: casticin increases the miR-338-3p expression in HL-60 (a) and THP-1 (b) cells in a dose-dependent manner. As shown in Figure S1, THP-1 cells were treated with different concentrations of casticin (1, 2, 4, 8 mg/mL), and the results showed that there was a significant decline in cell viability (Figure S1A), and the impact of casticin on cell viability was in a dose- and time-dependent manner. Subsequently, flow cytometry was performed to analyze the apoptosis rate of THP-1 in response to casticin treatment, which demonstrated a dramatic increase in the proportion of apoptotic cells in parallel with the augmented casticin concentration (Figure S1B). Additionally, we found that the number of TUNEL-stained THP-1 cells was increased when cells were treated with higher concentration of casticin (Figure S1C), consistent with results in Figure S1B. As shown in Figure S1D, miR-338-3p was suppressed in THP-1 cell compared with the normal bone marrow cells but obviously upregulated after exposure to casticin. As shown in Figure S2, miR-338-3p was obviously upregulated after exposure to casticin in HL-60 and THP-1 cells (*P* < 0.01), which was dose-dependent.

## References

[1] Short N. J., Ravandi F. (2016). Acute myeloid leukemia: past, present, and prospects for the future. *Clinical Lymphoma, Myeloma & Leukemia*.

[2] Short N. J., Rytting M. E., Cortes J. E. (2018). Acute myeloid leukaemia. *The Lancet*.

[3] Döhner H., Weisdorf D. J., Bloomfield C. D. (2015). Acute myeloid leukemia. *The New England Journal of Medicine*.

[4] Schiffer C. A. (2003). Hematopoietic growth factors and the future of therapeutic research on acute myeloid leukemia. *The New England Journal of Medicine*.

[5] Song L., Li X., Bai X. X., Gao J., Wang C. Y. (2017). Calycosin improves cognitive function in a transgenic mouse model of Alzheimer's disease by activating the protein kinase C pathway. *Neural Regeneration Research*.

[6] Wen Z., Hou W., Wu W. (2018). 6-O-Galloylpaeoniflorin attenuates cerebral ischemia reperfusion-induced neuroinflammation and oxidative stress via PI3K/Akt/Nrf2 activation. *Oxidative Medicine and Cellular Longevity*.

[7] Gao J., Song L., Xia H., Peng L., Wen Z. (2020). 6'-O-galloylpaeoniflorin regulates proliferation and metastasis of non-small cell lung cancer through AMPK/miR-299-5p/ATF2 axis. *Respiratory Research*.

[8] Zhu Y., Wang C., Luo J. (2021). The protective role of Zingerone in a murine asthma model via activation of the AMPK/Nrf2/HO-1 pathway. *Food & Function*.

[9] Wang C., Luo J., Bai X. (2021). Calycosin alleviates injury in airway epithelial cells caused by PM 2.5 exposure via activation of AMPK signalling. *Evidence-based Complementary and Alternative Medicine*.

[10] Lin S., Zhang H., Han T., Wu J. Z., Rahman K., Qin L. P. (2007). In vivo effect of casticin on acute inflammation. *Zhong Xi Yi Jie He Xue Bao*.

[11] Shiue Y. W., Lu C. C., Hsiao Y. P. (2016). Casticin induced apoptosis in A375.S2 human melanoma cells through the inhibition of NF-*κ*B and mitochondria-dependent pathwaysin vitroand inhibited human melanoma xenografts in a mouse modelin vivo. *The American Journal of Chinese Medicine*.

[12] Rasul A., Bao R., Malhi M. (2013). Induction of apoptosis by costunolide in bladder cancer cells is mediated through ros generation and mitochondrial dysfunction. *Molecules*.

[13] Zeng F., Tian L., Liu F., Cao J., Quan M., Sheng X. (2012). Induction of apoptosis by casticin in cervical cancer cells: reactive oxygen species-dependent sustained activation of Jun N-terminal kinase. *Acta Biochimica et Biophysica Sinica*.

[14] Haidara K., Zamir L., Shi Q. W., Batist G. (2006). The flavonoid casticin has multiple mechanisms of tumor cytotoxicity action. *Cancer Letters*.

[15] Tang S. Y., Zhong M. Z., Yuan G. J. (2013). Casticin, a flavonoid, potentiates TRAIL-induced apoptosis through modulation of anti-apoptotic proteins and death receptor 5 in colon cancer cells. *Oncology Reports*.

[16] Xia J., Gao J., Inagaki Y., Kokudo N., Nakata M., Tang W. (2013). Flavonoids as potential anti-hepatocellular carcinoma agents: recent approaches using HepG2 cell line. *Drug Discoveries & Therapeutics*.

[17] Ling Y., Zhu J., Fan M., Wu B., Qin L., Huang C. (2012). Metabolism studies of casticin in rats using HPLC-ESI-MSn. *Biomedical Chromatography*.

[18] Shen J. K., Du HP Y. M., Wang Y. G., Jin J. (2009). Casticin induces leukemic cell death through apoptosis and mitotic catastrophe. *Annals of Hematology*.

[19] Wang H. Y., Cai B., Cui C. B., Zhang D. Y., Yang B. F. (2005). Vitexicarpin, a flavonoid from Vitex trifolia L., induces apoptosis in K562 cells via mitochondria-controlled apoptotic pathway. *Yao Xue Xue Bao*.

[20] Papa V., Tazzari P. L., Chiarini F. (2008). Proapoptotic activity and chemosensitizing effect of the novel Akt inhibitor perifosine in acute myelogenous leukemia cells. *Leukemia*.

[21] Loges S., Tinnefeld H., Metzner A. (2006). Downregulation of VEGF-A, STAT5 and AKT in acute myeloid leukemia blasts of patients treated with SU5416. *Leukemia & Lymphoma*.

[22] He L., Hannon G. J. (2004). MicroRNAs: small RNAs with a big role in gene regulation. *Nature Reviews. Genetics*.

[23] Khalaj M., Tavakkoli M., Stranahan A. W., Park C. Y. (2014). Pathogenic microRNAâ€™s in myeloid malignancies. *Frontiers in Genetics*.

[24] Yeh C. H., Moles R., Nicot C. (2016). Clinical significance of microRNAs in chronic and acute human leukemia. *Molecular Cancer*.

[25] Fu L., Qi J., Gao X. (2019). High expression of miR-338 is associated with poor prognosis in acute myeloid leukemia undergoing chemotherapy. *Journal of Cellular Physiology*.

[26] Chen X., Pan M., Han L., Lu H., Hao X., Dong Q. (2013). miR-338-3p suppresses neuroblastoma proliferation, invasion and migration through targeting PREX2a. *FEBS Letters*.

[27] Guo B., Liu L., Yao J. (2014). miR-338-3p suppresses gastric cancer progression through a PTEN-AKT axis by targeting P-REX2a. *Molecular Cancer Research*.

[28] Lu R., Yang Z., Xu G., Yu S. (2018). miR-338 modulates proliferation and autophagy by PI3K/AKT/mTOR signaling pathway in cervical cancer. *Biomedicine & Pharmacotherapy*.

[29] Wen C., Liu X., Ma H., Zhang W., Li H. (2015). miR-338-3p suppresses tumor growth of ovarian epithelial carcinoma by targeting Runx2. *International Journal of Oncology*.

[30] Cohen-Solal K. A., Boregowda R. K., Lasfar A. (2015). RUNX2 and the PI3K/AKT axis reciprocal activation as a driving force for tumor progression. *Molecular Cancer*.

