# Supplementary material for: miR-338-3p Plays a Significant Role in Casticin-Induced Suppression of Acute Myeloid Leukemia via Targeting PI3K/Akt Pathway
Source: Biomed Res Int. 2022 Jun 18;2022:9214130. doi: 10.1155/2022/9214130 (PMC9233736; doi:10.1155/2022/9214130)
Supplement: Supplementary 1 — Figure S1: casticin inhibits THP-1 cell proliferation and promotes apoptosis. (a) THP-1 cells were treated with different concentrations of casticin (1, 2, 4, 8 mg/mL) and subjected to CCK-8 analysis at the indicated time intervals. Compared with the DMSO control group, ∗ indicates P < 0.05; ∗∗ indicates P < 0.01. (b) After treatment with casticin for 72 h, cell apoptosis was analyzed by performing flow cytometry. (c) Verification of the flow cytometry results by TUNEL assay. ∗P < 0.05; ∗∗P < 0.01. (d) The miR-338-3p expression was measured by qRT-PCR in THP-1 cell. ∗∗P < 0.01. [file 9214130.f1.docx]

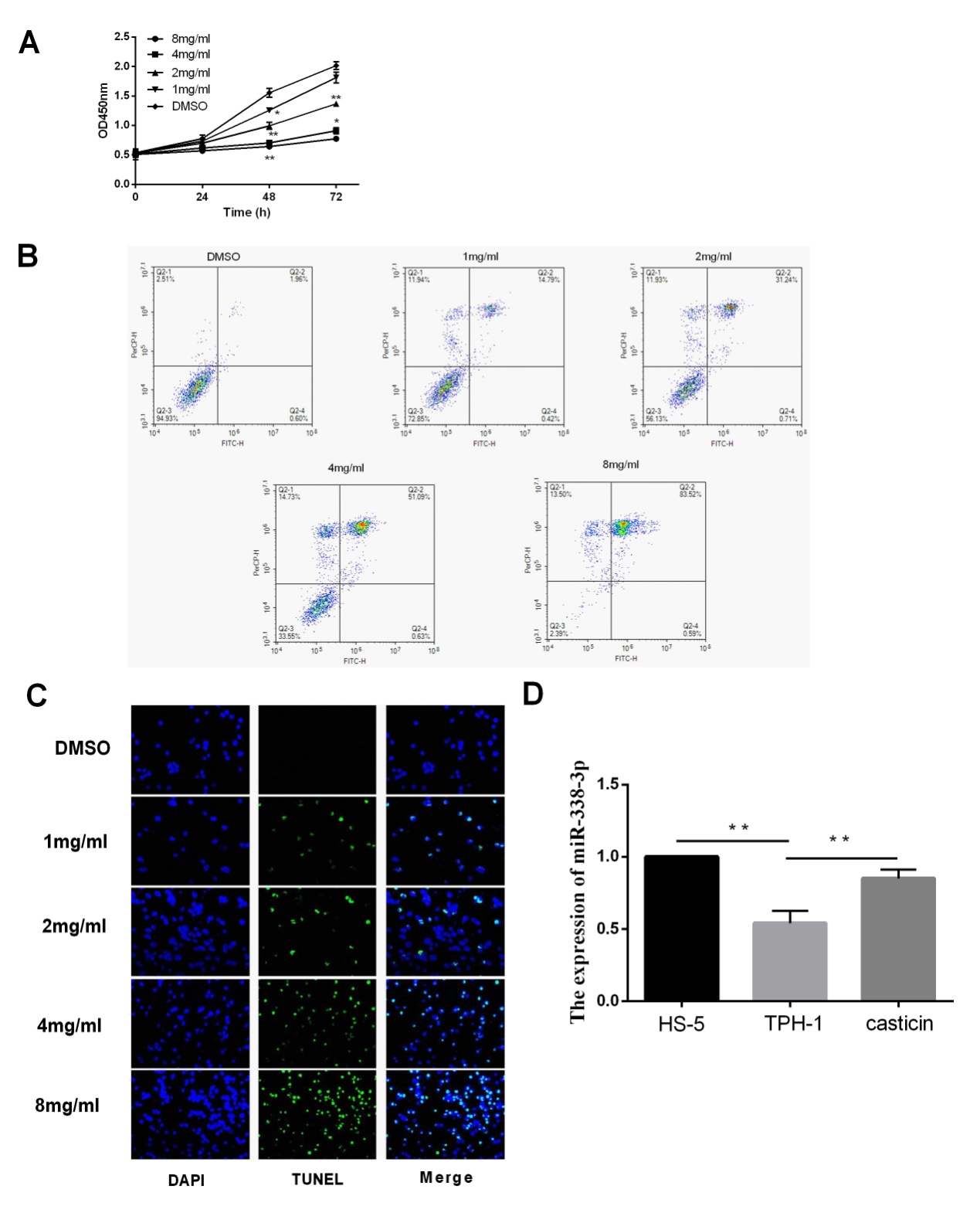


**Figure S1.** **Casticin inhibits THP-1 cell proliferation and promotes apoptosis.** (A) THP-1 cells were treated with different concentrations of casticin (1, 2, 4, 8 mg/mL) and subjected to CCK-8 analysis at the indicated time intervals. Compared with the DMSO control group, * indicates P<0.05; ** indicates P<0.01. (B) After treatment with casticin for 72 h, cell apoptosis was analyzed by performing flow cytometry. (C) Verification of the flow cytometry results by TUNEL assay. * P<0.05; ** P<0.01.
