# Supplementary material for: miR-338-3p Plays a Significant Role in Casticin-Induced Suppression of Acute Myeloid Leukemia via Targeting PI3K/Akt Pathway
Source: Biomed Res Int. 2022 Jun 18;2022:9214130. doi: 10.1155/2022/9214130 (PMC9233736; doi:10.1155/2022/9214130)
Supplement: Supplementary 2 — Figure S2: casticin increases the miR-338-3p expression in HL-60 (a) and THP-1 (b) cells in a dose-dependent manner. As shown in Figure S1, THP-1 cells were treated with different concentrations of casticin (1, 2, 4, 8 mg/mL), and the results showed that there was a significant decline in cell viability (Figure S1A), and the impact of casticin on cell viability was in a dose- and time-dependent manner. Subsequently, flow cytometry was performed to analyze the apoptosis rate of THP-1 in response to casticin treatment, which demonstrated a dramatic increase in the proportion of apoptotic cells in parallel with the augmented casticin concentration (Figure S1B). Additionally, we found that the number of TUNEL-stained THP-1 cells was increased when cells were treated with higher concentration of casticin (Figure S1C), consistent with results in Figure S1B. As shown in Figure S1D, miR-338-3p was suppressed in THP-1 cell compared with the normal bone marrow cells but obviously upregulated after exposure to casticin. As shown in Figure S2, miR-338-3p was obviously upregulated after exposure to casticin in HL-60 and THP-1 cells (P < 0.01), which was dose-dependent. [file 9214130.f2.docx]

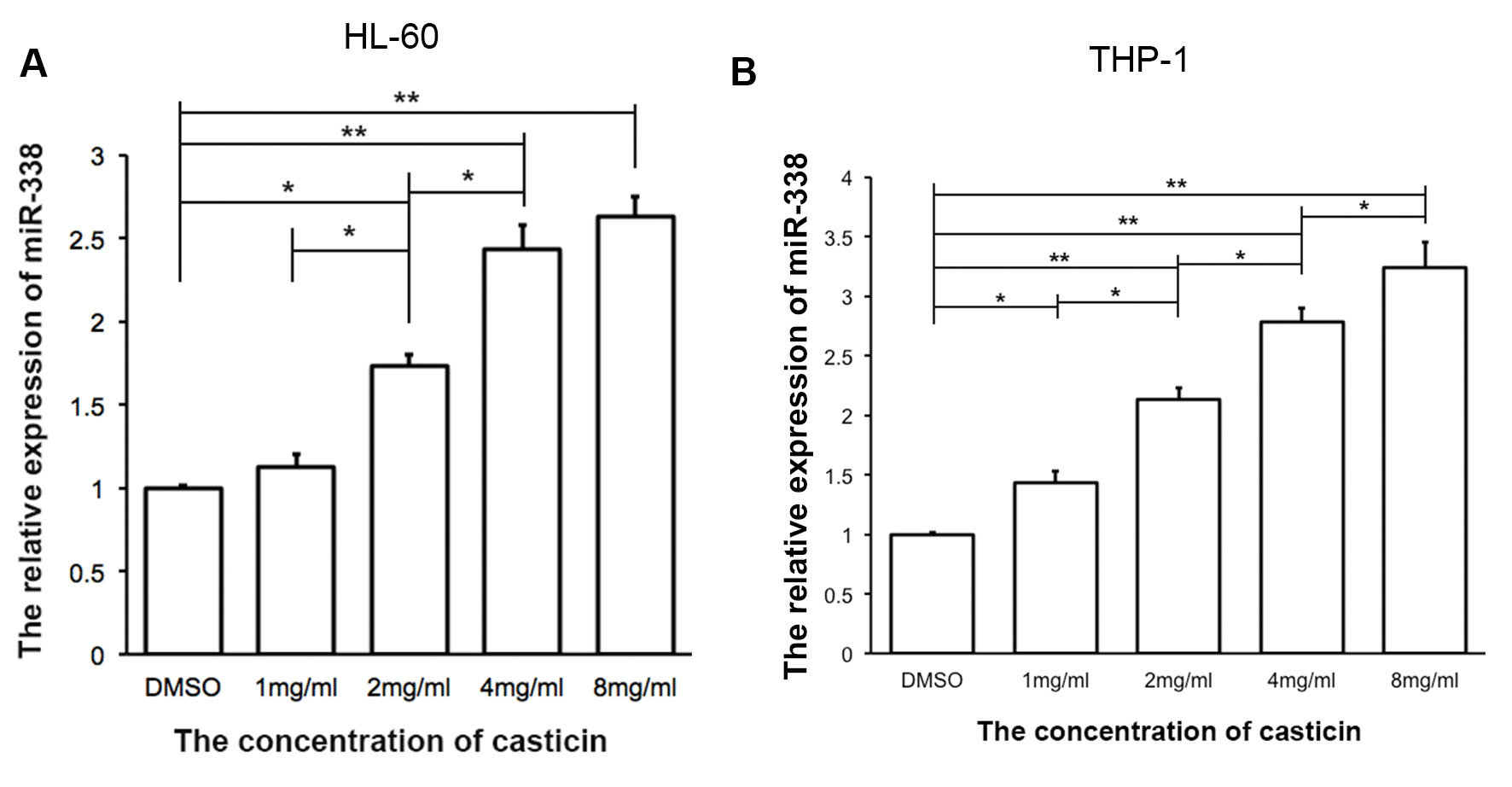


**Figure S2.** **Casticin increases the miR-338-3p expression in HL-60 (A) and THP-1 (B) cells in a dose-dependent manner.**
